# A New Approach to Oxidative Stress and Inflammatory Signaling during Labour in Healthy Mothers and Neonates

**DOI:** 10.1155/2015/178536

**Published:** 2015-02-01

**Authors:** Javier Díaz-Castro, Jesus Florido, Naroa Kajarabille, Sonia Prados, Catalina de Paco, Olga Ocon, Mario Pulido-Moran, Julio J. Ochoa

**Affiliations:** ^1^Department of Physiology, University of Granada, 18071 Granada, Spain; ^2^Institute of Nutrition and Food Technology “José Mataix”, University of Granada, 18071 Granada, Spain; ^3^Department Obtetrics and Gynecology, School of Medicine, University of Granada, 18071 Granada, Spain; ^4^Service of Obstetrics and Gynecology, University Hospital San Cecilio, 18071 Granada, Spain

## Abstract

The objective of the current study was to investigate for the first time and simultaneously the oxidative stress and inflammatory signaling induced during the delivery in healthy mothers and their neonates. 56 mothers with normal gestational course and spontaneous delivery were selected. Blood samples were taken from mother (before and after delivery) both from vein and artery of umbilical cord. Lower antioxidant enzymes activities were observed in neonates compared with their mothers and lower oxidative stress in umbilical cord artery with respect to vein. There was an overexpression of inflammatory cytokines in the mother, such as IL-6 and TNF-*α*, and, in addition, PGE_2_ was also increased. Neonates showed lower levels of IL-6 and TNF-*α* and higher values of sTNF-RII and PGE_2_ in comparison with their mothers. Parturition increases oxidative damage in the mother, although the indicators of oxidative damage were lower in umbilical cord artery with respect to umbilical vein. The overexpression of inflammatory cytokines reveals that fetus suffers its own inflammatory process during parturition.

## 1. Introduction

Several studies reported that parturition involves a strong oxidative stress for both mother and neonate implying an increased production of free radicals that must be controlled by their antioxidant system. This fact could lead to several functional alterations with important repercussion for the organisms that in the case of premature infants, by several reasons, are more acute [1–4].

There are different causes for this strong oxidative stress induced during parturition. Oxygen consumption is increased during pregnancy and parturition and therefore, as negative consequence, there is an increase in mitochondrial respiration and loss of electrons produced in the electron transport chain, facts that result in the formation of reactive oxygen species (ROS) [5–7]. In addition, during the progression of normal labor, powerful contractions of the myometrium and the associated increase in intrauterine pressure periodically suppress uteroplacental blood flow [8], giving rise to alternating cycles of ischemia and reperfusion [9]. The rapid change from relative hypoxic intrauterine to the extrauterine environment, where alveolar pO_2_ is almost five times higher, and the mediation of several physiologic processes involved in the finalization of the gestation and delivery are other reasons for this situation of oxidative stress [1–3].

Another important factor contributing to the increase in ROS production is the evoked inflammation during the delivery. Parturition has been identified as a source of proinflammatory mediators such as metabolites of arachidonic acid (prostaglandin E_2_, PGE_2_) and cytokines, including TNF-*α* and IL-6. These mediators are potent stimulators for the production of ROS and in turn free radicals recruit inflammatory signalers in a vicious circle [7, 10].

The knowledge of the antioxidant/oxidative status in normal pregnancy could be of help to better understand the physiological mechanisms involved in the diseases associated with pregnancy and to better indicate possible therapeutic targets. However, despite the importance of the mentioned aspects, the knowledge gained on this issue is still very limited in certain aspects. There are scarce studies aimed at assessing the relationship between the oxidative status of the mother and the newborn in healthy conditions [11]; moreover these studies are focused in plasma parameters, which can be easily modified by external factors [12, 13] and not in erythrocyte oxidative stress parameters that are more stable and accurate.

The majority of the available studies about oxidative stress and inflammation are focused on neonates with different pathologies or preterm babies, which limits the global knowledge of the factors involved in the healthy childbirth. On the other hand, many studies feature only a partial view of this process; for example, they discuss and feature only samples from umbilical cord vein [3, 4] or from vein and artery in the neonate but not in the mother [14]. In addition, some studies of the enzymatic antioxidant system in the neonate have aroused some controversy, partly because they just analyze the activity of just a single antioxidant enzyme [15]. Finally, another key factor to be taken into account is the lack of information about the processes taking place during labor in the mother. In particular, no data on the trend of the blood oxidative stress indicators are available at the parturition time. Nevertheless, a deeper knowledge of this multifactorial physiological process appears to be relevant. Therefore, in view of these considerations, this study was designed to assess for the first time and simultaneously the inflammatory process and oxidative stress associated with the delivery in healthy women and their neonates throughout the parturition, to have a clearer and complete view of this complex process.

## 2. Materials and Methods

### 2.1. Subjects

The study was performed on 56 mothers with normal gestational course and spontaneous onset of labor followed by normal delivery. Mean age was 29.9 ± 0.64 years, and mean gestational age was 39.3 ± 0.2 weeks. Parity was recorded as nulliparous (54.2%) or multiparous (45.8%). Newborn sexes were 50.9% male and 49.1% female. 52.5% of the deliveries were performed with epidural anesthesia and the 47.5% remaining without anesthesia. The inclusion criteria were absence of disease, singleton gestation, normal course of pregnancy, term gestation with cephalic presentation, body mass index of 18–30 kg/m^2^ at the start of pregnancy, weight gain of 8–12 kg since pregnancy onset, gestational age at delivery of 38–42 weeks, spontaneous vaginal delivery, newborn with appropriate weight for gestational age, newborn with Apgar index ≥7 at 1st and 5th min of life, and normal monitoring results. Progress of labor was determined by vaginal examinations every one to two hours and as indicated by clinical conditions. Uterine contractions and fetal heart rate were monitored continuously with a cardiotocograph and were normal in all the cases. None of these subjects showed any abnormalities during labor and delivered spontaneously. The maternal-fetal ejection period lasted 45.2 ± 5.5 min, in all the subjects. The study was approved by the Bioethical Committee on Research Involving Human Subjects at the University Hospital “Virgen de las Nieves” in Granada, and consent was obtained from the parents after the nature and purpose of the study had been explained to them and were fully understood.

### 2.2. Blood Sampling

Maternal blood samples were obtained from the antecubital vein at two different times: at the beginning of the active phase of labor and at the start of expulsion when the fetus was at station +2. From the umbilical cord, blood samples were collected from vein and artery, immediately after cord clamping. During the examination, the size, shape, consistency, and completeness of the placenta were determined, and the absence of accessory lobes, placental infarcts, hemorrhage, tumors, and nodules was recorded. Blood was immediately centrifuged at 1750 ×g for 10 min at 4°C in a Beckman GS-6R refrigerated centrifuge (Beckman, Fullerton, CA, USA) to separate plasma from red blood cell pellets. Plasma samples were immediately frozen and stored at −80°C until analysis. Erythrocyte cytosolic and membrane fractions were prepared by differential centrifugation with hypotonic hemolysis and successive differential centrifugations according to the method of Hanahan and Ekholm [16]. The final fractions were aliquoted, snap-frozen in liquid nitrogen, and stored at −80°C until analysis.

### 2.3. Biochemical Parameters

Total bilirubin, triglycerides, total cholesterol, and phospholipids were determined using a commercial kit (Spinreact, Barcelona, Spain) and following the instructions of the manufacturer.

### 2.4. Inflammatory Parameters

Tumor necrosis factor-*α* (TNF-*α*), interleukin-6 (IL-6), and soluble receptor II of TNF-*α* (sTNF-RII) plasma levels were determined using Biosource kits (Biosource Europe, Nivelles, Belgium), PGE_2_ was determined using a R&D kit (R&D Systems Europe, Abingdon, United Kingdom). The TNF-*α*, IL-6, and PGE_2_ are solid phase enzyme amplified sensitivity immunoassays (EASIA) performed on microtiter plate. After assays the samples were read at an appropriate wavelength (450–490 nm) on a microplate reader (Bio-Tek, Vermont, USA). The sTNF-RII kit is a solid phase sandwich enzyme linked immunosorbent assay (ELISA). The microtiter plate is then read at an appropriate wavelength (450 nm) on a Bio-Tek microplate reader (Bio-Tek, Vermont, USA).

### 2.5. Oxidative Stress Parameters

The antioxidant power of biological fluids can be evaluated either by quantification of individual enzymatic antioxidants or by assessing their aggregate, cumulative action and synergic effect. This latter concept is known as the total antioxidant status (TAS). Plasma TAS was analyzed with the use of the TAS Randox kit (Randox laboratories, Ltd, Crumlin, UK). The assay involves brief incubation of ABTS (2,2′-Azinobis-di[3-ethylbenzthiazoline sulfonate]) with peroxidase (metmyoglobin) and hydrogen peroxide, resulting in the generation of ABTS + radical cations. The method detects a reduction in the generation of the ABTS + radical cations by plasma antioxidants, and the decrease in the generation of ABTS + radical cations is proportional to their total antioxidant concentration. 200 *μ*L of chromogen (metmyoglobin and ABTS) and 4 *μ*L of plasma sample/standard control/distilled water were added, incubated at 37°C for 10 s, and read at 630 nm. This was followed by the addition of 40 *μ*L of substrate (hydrogen peroxide in stabilized form), incubation at 37°C for exactly 3 min, and measuring absorbance at 630 nm. A standard control (6-hydroxy-2,5,7,8-tetramethylchroman-2-carboxylic acid) was provided in the kit. Results were expressed in mM of Trolox equivalents. The linearity of calibration extends to 2.5 mmol/L of Trolox. The reference range for human blood plasma is given by the manufacturer as 1.30–1.77 mmol/L. Measurements in duplicate were used to calculate intra-assay variability. For glutathione peroxidase (GPx), we used the technique of Flohé and Günzler [17], a method based on the instantaneous formation of oxidized glutathione during the reaction catalysed by glutathione peroxidase. During the reaction, cumene hydroperoxide was used as substrate. Catalase (CAT) activity was determined following the method described by Aebi [18], monitoring at 240 nm spectrophotometrically (Thermo Spectronic, Rochester, USA) the H_2_O_2_ decomposition, as a consequence of the catalytic activity of CAT. The activity was calculated from the first-order rate constant *K* (sec^−1^). Superoxide dismutase (SOD) was determined by the method of Crapo et al. [19], based on the inhibition by SOD of the reduction of cytochrome C, measured by spectrophotometry at 550 nm (Thermo Spectronic, Rochester, USA). Plasma hydroperoxides were determined using the OxyStat kit (Biomedica Gruppe, Vienna, Austria). OxyStat is a colorimetric assay for the quantitative determination of peroxides in plasma, serum, and other biological fluids. The peroxide concentration is determined by reaction of the biological peroxides and a subsequent color reaction using TMB (3,3′,5,5′-tetramethylbenzidine) as substrate. The plate was measured at 450 nm wavelength on a Bio-Tek microplate reader (Bio-Tek, Vermont, USA). Erythrocyte membrane hydroperoxides were estimated by the method described previously [20]. The method is based on the principle of the rapid peroxide-mediated oxidation of Fe^2+^ to Fe^3+^ under acidic conditions. The latter, in the presence of xylenol orange, forms a Fe^3+^-xylenol orange complex which can be measured spectrophotometrically at 560 nm wavelength (Perkin Elmer UV-VIS Lambda-16, Norwalk, Connecticut, USA).

### 2.6. Statistical Analysis

Results are presented as mean ± SEM. Before any statistical analysis, all variables were checked to assess their normality using the Levene test. When a variable was found to be not normal it was transformed to develop the statistical analysis. All the results were submitted to one-way ANOVA analysis. Bonferroni's test was performed* a posteriori* to evaluate differences between groups. All *P* values of 0.05 or less were considered significant. SPSS version 18.0, 2010 (SPSS Inc., Chicago, IL, USA), software has been used for data treatment and statistical analysis.

## 3. Results

The delivery involves diverse modifications in the plasmatic biomarkers, some of which are shown in Table 1.With respect to bilirubin, we observed a statistical increase in the umbilical vein compared with the artery (*P* < 0.05) (Table 1).

It is noteworthy the effect of pregnancy and parturition on the plasmatic lipids studied. In our study, we found higher values of plasmatic total cholesterol, phospholipids, and triglycerides in the mother than in the umbilical cord (*P* < 0.05). During parturition only plasmatic phospholipids decreased in the mother (*P* < 0.05) and we found higher values of total cholesterol (*P* < 0.001) and phospholipids (*P* < 0.05) in umbilical vein in comparison with the umbilical artery (Table 1). Related to level of triglycerides, no differences were checked between mothers before and after delivery and umbilical cord, both vein and artery.

We observed a decrease in plasma peroxides (*P* < 0.05) and membrane erythrocyte hydroperoxides in umbilical artery compared with those from umbilical vein (*P* < 0.05). In addition, an increase in both plasma peroxides and membrane erythrocyte hydroperoxides were found in the mother during parturition (*P* < 0.05) (Table 2).

With regard to the antioxidant system (Table 2), the results showed that the mothers suffer a decrease in plasmatic TAS during the delivery (*P* < 0.05). However, CAT and SOD activities were not modified during the delivery process for both mother and neonates. On the other hand, both CAT and GPx showed lower values in the neonates (both vein and artery) compared with the mothers before and after delivery (*P* < 0.05). With regard to the differences found between umbilical vein and artery, only TAS showed a lower value in vein compared with artery (*P* < 0.05).

**Figure 1:**
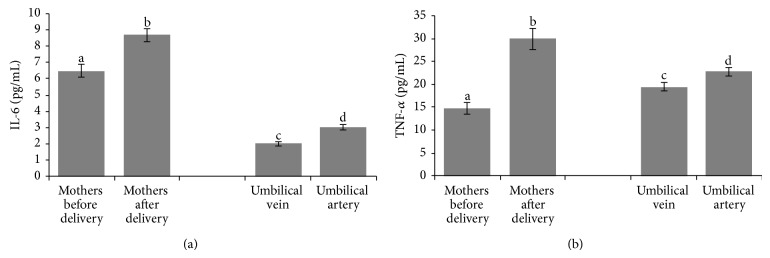
Values of IL-6 (a) and TNF-*α* (b) in plasma from mothers (*n* = 56) (before and after delivery) from both vein and artery of umbilical cord. Values are mean ± SEM. Columns with different superscript letters were significantly different (*P* < 0.05).

**Figure 2:**
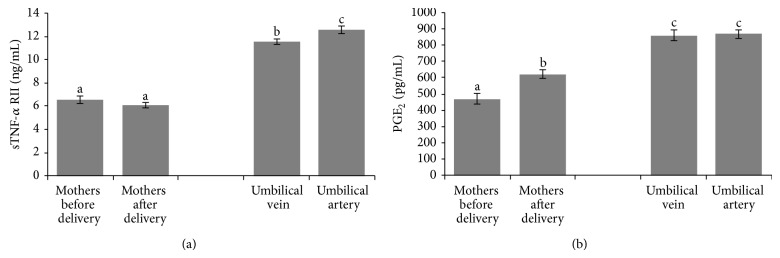
Values of sTNF-RII (a) and PGE-2 (b) in plasma from mothers (*n* = 56) (before and after delivery) from both vein and artery of umbilical cord. Values are mean ± SEM. Columns with different superscript letters were significantly different (*P* < 0.05).

In addition, parturition leads to an overexpression of inflammatory cytokines such as IL-6 (*P* < 0.001) (Figure 1(a)), TNF-*α* (Figure 1(b)) (*P* < 0.001), and PGE_2_ (*P* < 0.001) (Figure 2(b)) in the mother, meanwhile sTNF-RII did not show any modification (Figure 2(a)). We have observed higher values of PGE_2_ and sTNF-RII in blood from umbilical cord (both in vein and artery) (Figures 2(b) and 2(a), resp.) than those found in the mother, before and after delivery (*P* < 0.001 and *P* < 0.05, resp.). IL-6 was lower in umbilical cord (vein and artery) in comparison with the mother (*P* < 0.001) and TNF-*α* showed higher concentrations in umbilical cord artery and vein than in mother's blood at the beginning of dilatation; however lower concentrations were recorded in umbilical cord artery and vein than in mother's blood before the maternal-fetal ejection (Figure 1). With respect to the differences between umbilical vein and artery, the results showed higher values of IL-6, TNF-*α*, and sTNF-RII in artery than in vein (*P* < 0.05) (Figures 1 and 2).

## 4. Discussion

Many aspects about oxidative stress and inflammation during parturition are still not completely elucidated. Therefore, a complete approach to these processes both in the mother and the newborn is necessary. Taking into account this scenario, the present study was designed to simultaneously assess, both in mothers and newborns, variations in oxidative stress status, inflammatory signalling, and biochemical parameters occurring in blood samples during the course of the delivery.

In our case, to have a solid baseline, blood samples of mothers were taken from the antecubital vein, at the beginning of the cervix dilatation and again immediately before the maternal-fetal ejection. Also, blood samples were collected from the umbilical vein and arteries. Taking a sample from each blood type, we can assess which mediators are transferred to the fetus from the mother or produced by the fetus, showing the key role of placental barrier.

Many studies have showed antioxidant effects of bilirubin, even higher than those shown for vitamin E [21]. Therefore, the higher bilirubin maternal-fetal transfer could help the newborn to avoid, at least in part, the oxidative stress induced, acting as a compensatory mechanism induced by the labor. With respect to its origin, there are many studied factors demonstrating their influence on bilirubin levels. One of the key factors which influence bilirubin levels is the oxytocin, a hormone that is increased during labor induction [22, 23].

Increases in serum lipids are commonly reported during the second half of pregnancy [24] and could be related, at least in part, with pregnancy hormones and the stress induced by the delivery [25–27]. Anyway, this maternal hyperlipidaemia could have a beneficial influence on fetal development [24], because as our results show, the mother transfers more cholesterol and phospholipids to the fetus through the vein than the fetus transfers to the mother through the umbilical artery, indicating an uptake by the fetus, probably due to high necessity of these molecules for the fetus development [24].

Potential sources of ROS during the parturition include the mother, the placenta, and the fetus [28]. Trying to have a complete view of the phenomena, we have studied two of them, the mother and the fetus. If the fetus were the main source of ROS, then the umbilical cord artery ROS levels would be expected to be higher than umbilical vein. However, we could observe a decrease in plasma and membrane erythrocyte hydroperoxides in umbilical artery compared with those from umbilical vein. These findings suggest that the fetus metabolizes, rather than generating those free radicals [28]. Another potential source of free radicals is the mother. Parturition implies a strong oxidative aggression to the mother, a fact that can be deduced from the results showing an increase in both plasma peroxides and membrane erythrocyte hydroperoxides in the mother during parturition. Part of this aggression could be targeted to the fetus through the umbilical vein, a fact that explains the higher values in umbilical vein than in artery. In this sense, a good correlation has been shown between the oxidative stress of the mother and the neonates, showing that a high oxidative stress in the mother also induces high oxidative stress in umbilical vein [11].

With regard to the antioxidant system, the results are in agreement with the information featured by the oxidative damage; therefore the mothers suffer a decrease in plasmatic TAS during the delivery, due to its reduction in the process of neutralization free radicals generated during the labor, compensating the higher oxidative damage. This result can be directly linked with the higher values of GPx to compensate the oxidative aggression in erythrocyte membranes of the mothers after delivery [29, 30]. A different behaviour is featured by CAT and SOD, whose activities were not modified during the delivery process. The unchanged levels of SOD that we observed are in agreement with the results of Telfer et al. [31]. Those findings led them to postulate that SOD may have other roles to play in the implantation and maintenance of uterine quiescence. In addition, Sugino et al. [32] have suggested that SOD is steroidally regulated and that decreased activity of this enzyme is implicated in failed pregnancies. It is important to consider that this enzyme plays a crucial role in the antioxidant defence; in addition during the delivery and in the neonate a high activity of the xanthine/xanthine oxidase system is featured and therefore a high production of anion superoxide [4]. Therefore, during the whole process of the delivery, high activities of this enzyme are recorded, probably to compensate the high generation of superoxide anion, together with an unchanged activity of CAT, an enzyme that acts together with SOD neutralizing and scavenging free radicals. The importance of SOD and xanthine/xanthine oxidase system as enzymatic defence against free radicals during delivery is supported by the information obtained in samples of umbilical cord, because SOD is the only enzyme that shows a similar activity to those values found in the mother, both in umbilical vein and artery. On the other hand, both CAT and GPx showed lower values in neonates compared with the mothers; however, due to the lack of data existing in the scientific literature, this result is difficult to be fully explained, although we think that can be due to the utilization of both enzymes in the neutralization of free radicals during the labor and to the natural lower levels of plasma peroxides described previously. With regard to the differences found between umbilical vein and artery, only TAS showed a lower value in vein than in artery, a fact that can be linked with a major use of these antioxidants defences due to a higher content of plasma peroxides. In general, this information, together with the lower plasma peroxides and membrane erythrocyte hydroperoxides in the umbilical artery mentioned above, indicates that the neonate has enough antioxidant capacity to scavenge and metabolize the production of free radicals during the labour.

Many mechanisms underlying the onset of labour remain unclear, although there is increasing evidence pointing to the inflammatory nature of human parturition [33]. Parturition compromises of five separate but integrated physiological events and increasingly evidence supports a role for prostaglandins and cytokines in each of these events [34]. In this sense, the uterus is activated by proinflammatory cytokines and between other actions, one of their roles is the stimulation of prostaglandin (PG) synthesis which are potent stimulators of myometrial contractility and critical factors in the initiation of the labour [34–37]. However, as occurs with oxidative stress, our understanding of the specific contributions of these inflammatory signalers during pregnancy remains relatively limited.

In the current study, parturition leads to a remarkable overexpression of inflammatory cytokines such as IL-6, TNF-*α*, and PGE_2_ in the mother; meanwhile sTNF-RII did not show any modification. The inflammatory signaling relationship between mother and infant has not been studied extensively and therefore our study reports a more complete view of the physiological process. Some studies reported high concentrations of IL-6 [38] and TNF-*α* after the onset of labour [39], data that are in agreement with our results. Part of this IL-6 seems to be generated by the placenta [40] which also releases TNF [41]. TNF-*α* is also increased in amnion, chorion, and decidua with labour at term [42]. Our results also show that placenta acts as a barrier for these proinflammatory cytokines, showing lower values in umbilical cord blood than in the mother's blood before maternal-fetal ejection. Other interesting results found in the current study reveal that the fetus suffer its own inflammation process with higher values of IL-6 and TNF-*α* in umbilical artery that those found in vein. However, in the same way, the fetus is able to produce anti-inflammatory cytokines to balance and face the inflammatory process, and in this sense, a higher value of sTNF-RII in umbilical artery was found compared with umbilical vein. This finding reveals that the increase in sTNF-RII reduces the detrimental, proinflammatory effects of TNF-*α* in the fetus. sTNFR-II stimulation has revealed activation of the immunosuppressive IL-10 pathway and inhibits significantly the effects of several proinflammatory cytokines [43]. Placenta uses a similar mechanism to reduce the negative effect of TNF-*α* in the fetus, because overexpression of sTNF-RII in placenta has been observed with the aim to sequester TNF [44]. This fact could also explain the higher values of sTNF-RII in umbilical vein compared with those found in the mother's blood.

Another interesting finding is the results of PGE_2_. Parturition is associated with increased PG levels in different compartments and sources [34, 45–48], although the fetus is the main source, as we can deduce from the results obtained in the current study. It has been observed that PGE_2_ concentrations increase progressively in the fetal circulation over the last 15–20 days of gestation, associated with an increase in fetal plasma cortisol and consistent with the stimulatory effects of PGE_2_ on the fetal hypothalamic-pituitary-adrenal axis [47, 48]. This could explain the higher values of PGE_2_ found in umbilical artery. On the other hand, increased fetal cortisol regulates the expression of prostaglandin synthase type 2 in the placenta in an estrogen-independent manner, resulting in increased concentration of PGE, which could explain the values found in umbilical vein and also could in part be responsible for the increase in the values of PGE_2_ during parturition in the mother. Other possible sources of PGE_2_ in the mother are the proinflammatory cytokines [46] and the increase in the maternal uterine expression of prostaglandin G/H synthase 2 (PGHS-2) in an estrogen-dependent manner [48].

In summary, our results show an increase in oxidative damage mediators in the mother during parturition, with lower antioxidant enzymes activities in the neonates compared with their mothers and lower oxidative stress in the artery of umbilical cord with respect to vein. We also observed an overexpression of inflammatory cytokines in the mother during parturition, higher levels of IL-6 and TNF-*α* and sTNF-RII in the umbilical artery compared with the vein, a fact that indicates that the fetus suffers its own inflammatory process during parturition. Finally, our results give information about the origin of PGE_2_ during parturition and strengthen the important role of PGE_2_ from fetus in the process leading to birth. Therefore, this study supplies new information about the both immunological and oxidative stress relationship between mother and fetus, aspects that have not been extensively studied. These results are important because they add more information about the role of the inflammation and oxidative stress in normal human labour at term hinders attempts to prevent preterm birth or the treatment of diseases associated with pregnancy. However, further research is needed to elucidate this issue with a wide perspective and to complete the results obtained.

## Figures and Tables

**Table 1:** Values of biochemical parameters studied in mothers during labour and in both umbilical vein and artery of newborn.

	Mothers (*n* = 56)		Umbilical cord (*n* = 56)	
	Before delivery	After delivery	Vein	Artery
Total bilirubin (*µ*mol/L)	46.74 ± 4.20^a^	49.04 ± 4.35^a^	30.27 ± 1.52^b^	26.03 ± 1.36^c^
Total cholesterol (mg/dL)	257.66 ± 5.62^a^	242.01 ± 6.99^a^	65.21 ± 1.28^b^	60.25 ± 1.39^c^
Phospholipids (mg/dL)	194.56 ± 4.18^a^	182.68 ± 3.68^b^	97.02 ± 2.73^c^	89.32 ± 2.69^d^
Triglycerides (mg/dL)	197.85 ± 9.86^a^	185.82 ± 8.43^a^	44.08 ± 1.89^b^	44.41 ± 2.13^b^

Values are mean ± SEM. For every column, mean values with different superscript letters were significantly different (*P* < 0.05).

**Table 2:** Oxidative stress biomarkers in plasma and erythrocyte from mothers (before and after delivery) and from both vein and artery of umbilical cord.

	Mothers (*n* = 56)		Umbilical cord (*n* = 56)	
	Before delivery	After delivery	Vein	Artery
Plasma parameters				
TAS (nmol/mL)	1.06 ± 0.03^a^	0.98 ± 0.03^b^	0.96 ± 0.02^b^	1.07 ± 0.03^a^
Peroxides (nmol/mL)	9.93 ± 0.42^a^	11.11 ± 0.38^b^	7.89 ± 0.82^c^	6.70 ± 0.24^d^
Erythrocyte parameters				
CAT cytosol (K/seg·mg)	0.389 ± 0.021^a^	0.403 ± 0.016^a^	0.287 ± 0.011^b^	0.269 ± 0.009^b^
GPx cytosol (U/mg)	55.18 ± 2.87^a^	64.93 ± 2.67^b^	32.91 ± 1.06^c^	33.17 ± 1.21^c^
SOD cytosol (U/mg)	215.56 ± 9.01^a^	221.73 ± 8.23^a^	233.89 ± 6.96^a^	220.39 ± 7.89^a^
Membrane hydroperoxides (nmol/mL)	22.83 ± 1.13^a^	26.87 ± 1.38^b^	25.91 ± 0.99^b^	22.58 ± 0.95^a^

Values are mean ± SEM. For every column, mean values with different superscript letters were significantly different (*P* < 0.05).
